# Lactococcus garvieae in Rural Alabama: A Case Report

**DOI:** 10.7759/cureus.39560

**Published:** 2023-05-27

**Authors:** Bisma Masudi, Tetiana Litvinchuk, Jessica Byrd

**Affiliations:** 1 Osteopathic Medical Student-III, Alabama College of Osteopathic Medicine, Dothan, USA; 2 Pediatrics, Helen Keller Hospital, Sheffield, USA; 3 Pediatrics, Hellen Keller Hospital, Sheffield, USA

**Keywords:** water exposure, gram-positive bacteria, wound infections, lactococcus, zoonotic pathogens, lactococcus garvieae (l. garvieae)

## Abstract

*Lactococcus (L.) garvieae* is a gram-positive coccus that has been found in various aquatic and terrestrial animals, as well as in dairy products, and is considered a potential zoonotic bacterium. The pathogen has been recognized as an emerging opportunistic human pathogen, often associated with the ingestion of raw seafood. The most common presentation of *L. garvieae* infection in humans is infective endocarditis, but it has also been found to have associations with other clinical manifestations. The following is a case report of a 6-year-old male with infected bilateral leg abrasions that occurred after playing in a local creek near his home in northern Alabama, which had livestock including goats, cows, and horses. Wound culture indicated that the bacteria was *L. garvieae,* which was found to be sensitive to ceftriaxone, levofloxacin, linezolid, tetracycline, tigecycline, and vancomycin and resistant to clindamycin. The patient was treated with oral cephalexin and topical gentamicin for ten days, after which there appeared to be an overall improvement in wound healing.

## Introduction

*Lactococcus garvieae* is a gram-positive coccus that was originally classified as *Streptococcus garvieae* and has since been reclassified under the genus Lactococcus [1]. Currently, *L. garvieae* is one of 11 recognized species within the Lactococcus genus [2]. The bacteria is classified as a facultatively anaerobic, alpha-hemolytic, non-motile, and non-spore-forming gram-positive ovoid coccus that can be found in pairs and short chains [1].

*L. garvieae* is most commonly associated with warm-water fish infections and has been found to be responsible for lactococcosis or hemorrhagic sepsis in the farmed trout industry [1]. Since the first documented case in humans in 1991, *L. garvieae* has been increasingly recognized as an emerging opportunistic human pathogen [3]. Often associated with the ingestion of raw seafood, bacterial infections have been reported in many different countries [4]. In addition, the pathogen has also been isolated from vegetables, cereals, raw beef and pork [4,5]. It has also been noted to have been found as a naturally occurring component of many different dairy products, such as raw milk and artisan cheeses, and as such, there are numerous potential vectors for infection [4].

The potential zoonotic bacterium’s isolation from various aquatic and terrestrial animals, as well as from rivers and sewage waters, demonstrates its ability to adapt and survive in diverse and harsh environmental conditions [4,6]. The primary condition that it causes in human hosts is infective endocarditis, with many such reports noted in the literature [6]. Beyond infective endocarditis, *L. garvieae* has been found to have associations with liver abscesses, peritonitis, urinary infections, and infective spondylodiscitis [7,8,9,10,11]. It has been noted in very rare cases to also cause serious superficial infections, but there are very few examples noted in the literature [5]. Predisposing factors for *L. garvieae* infection include, but are not limited to, older age, cardiovascular and metabolic diseases, the presence of prosthetics, prior surgeries, and intestinal tract alterations [12]. The following case has been found to be relatively unique in the literature, as the method of transmission has not been well described. In this particular case, a healthy 6-year-old child was wading in a river with bilateral abrasions from his boots that were ultimately found to be infected by this bacteria. While *L. garvieae* is not a frequently identified causative agent of infection in humans, we hope this case reminds clinicians to always maintain a broad differential and, as such, always be open to further investigation. 

## Case presentation

**Figure 1:**
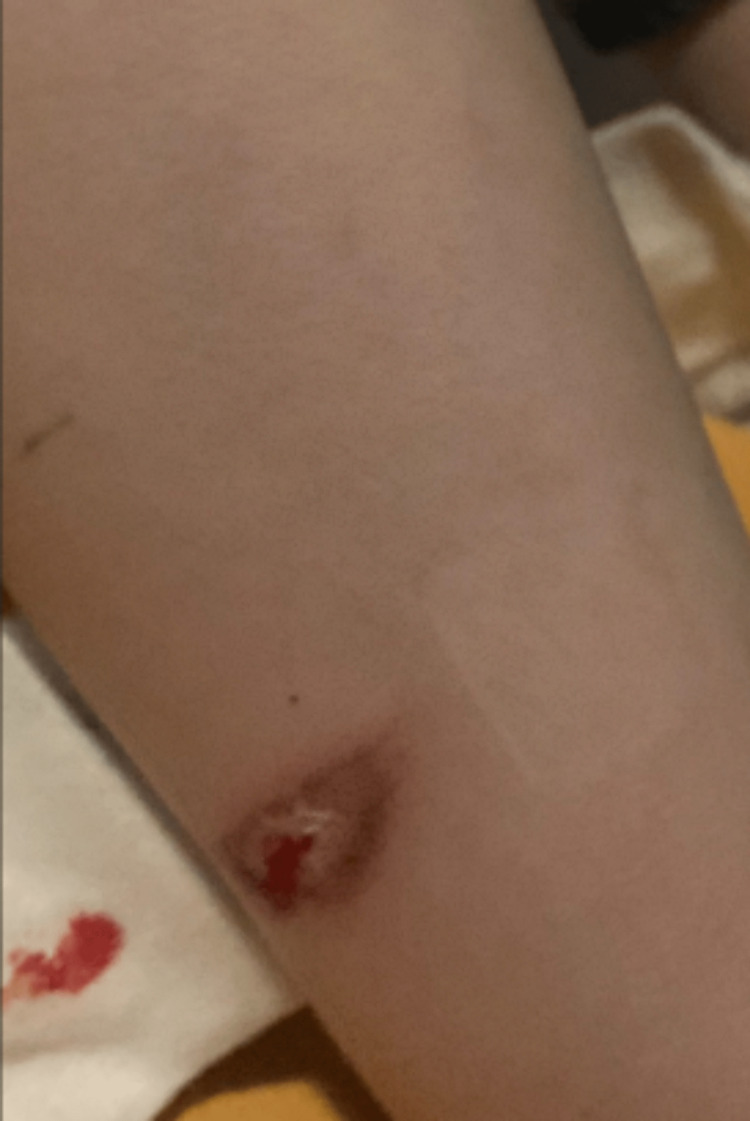
Initial appearance of one wound prior to bringing the patient to the clinic. Scrape lesion on the calf with bloody drainage.

The patient is a 6-year-old male who presented with bilateral leg abrasions that were acquired a few days prior to arrival with evident signs of infection, including erythema and drainage. Refer to Figure 1 for the initial appearance of one of the wounds when acquired. 

There were no additional symptoms of infection, including lethargy, fever, or associated pain. A thorough history revealed that he had recently been wading in a local creek in northern Alabama while wearing rubber boots that ultimately scraped against his legs, causing the aforementioned lesions. The creek in question is located near his family home, which houses livestock such as goats, cows, and horses. The patient had no significant medical history, and family history was non-contributory. The patient was fully up-to-date on immunizations. Vital signs were within normal limits, including a temperature of 97.1°F. At the time of the initial visit, abrasions were erythematous with circular impetigo-like bullae and a small amount of drainage from all of the wounds bilaterally.

A wound culture was obtained, and the patient’s parent was counseled to return to the office as needed or if the patient’s condition worsened. Symptomatic care, pain control with over-the-counter analgesics, and cephalexin 250 mg/5 mL oral suspension were prescribed based on clinical presentation. The parent was given instructions to dispense 5 mL of cephalexin oral suspension every six hours for ten days to the patient. Furthermore, mupirocin 2% topical ointment with two refills was also prescribed with instructions to apply it three times a day to the affected area for ten days.

**Figure 2:**
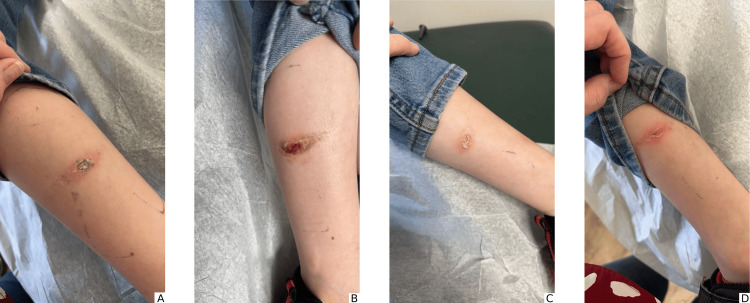
Presentation of wounds at the time of the second encounter: A. Dried scabbing with minor erythema on the posterior leg; B. Wound with dried bloody drainage on the lateral leg; C. Abrasion with residual scabbing on the lateral leg; D. Erythematous lesion with scaling on the posterior leg

Wound culture results arrived after four days, and it was revealed that the bacteria present in the wound was *Lactococcus garvieae*. In light of previous research indicating its sensitivity to amoxicillin, the medication was changed to half a teaspoon of amoxicillin/clavulanic acid (42.9 mg/5 mL) oral suspension twice a day for ten days [2]. Additionally, the mupirocin topical ointment was replaced with gentamicin 0.1% ointment, to be applied three times daily to the affected area for a period of ten days, as the literature has demonstrated the bacteria's sensitivity to gentamicin in other cases [2]. When the antibiotic sensitivity panel results arrived the following day, it was revealed that the pathogen was susceptible to ceftriaxone, levofloxacin, linezolid, tetracycline, tigecycline, and vancomycin. It was found to be intermediately sensitive to ampicillin, cefotaxime, and penicillin-g. Finally, the pathogen was also noted to be resistant to clindamycin. The patient returned to the clinic the next day following sensitivity panel results for a repeat evaluation of his bilateral lower extremity abrasions. At this time, he was placed back on cephalexin in addition to the gentamicin topical ointment. Since the initial encounter, the patient’s parents had reported an overall improvement in the patient’s wound healing and denied any febrile episodes. At this second encounter, five days following the initial presentation, wounds appeared as four 3.8 cm x 3.8 cm dry, scaly lesions on both sides of his lower legs, with mild bloody drainage from the wounds. No additional associated symptoms had developed during the course of the antibiotics, and the wounds were found to have reduced drainage and erythema compared to the initial encounter. The parent was counseled to use antibacterial soap daily and was told to call the clinic if the patient began to experience fever, abdominal pain, vomiting, or any other concerning symptoms. At the time of writing, the patient has fully healed and has had no residual complications from the infection. Refer to Figure 2 for patient presentation at the time of the second encounter.

## Discussion

While this case report of an admittedly rare pathogen means that it is less likely for other clinicians to also be exposed to patients who have been infected by it, the authors hope that this case report can provide some illumination on potential causes for infection beyond the most common culprits. We do, however, hope to provide some unique insight into *L. garvieae’s* ability to cause infection in a manner that is not through ingestion and is not directly facilitated by the consumption of any form of infected food. The patient’s infection via direct contamination indicates the importance of water safety and education in children and limiting exposure of open wounds to outside elements, as an infection could easily have been caused by a myriad of other pathogens as well. This case is unique in that the patient was a healthy child without any predisposing illnesses or any significant family history of cardiovascular or gastrointestinal disease, systems that have been found to play a role in *L. garvieae’s* extent of infection in previous studies [4,13]. Fortunately for the patient, the pathogen was found to be sensitive to ceftriaxone, levofloxacin, linezolid, tetracycline, tigecycline, and vancomycin medications and resistant to clindamycin. This pattern of general susceptibility to most broad-spectrum antibiotics but resistance to clindamycin has also been noted in prior infections in the literature [14]. Determining which specific medication to prescribe involves not only considering which antibiotics the bacteria are sensitive to but also the child’s age and what is feasible for a parent to give a child daily. As such, cephalexin was deemed the most appropriate due to the high risk associated with the use of fluoroquinolones and tetracyclines in children, the intermediate resistance of ampicillin and penicillin-g, as well as the intramuscular nature of ceftriaxone, increasing the risk of non-compliance.

It is also interesting to note that this case occurred in rural northern Alabama in early March when temperatures were cooler as opposed to the warm temperatures of June through August that *L. garvieae* usually becomes endemic in [3]. Although its optimal growth temperature is 37°C, it can grow between 10°C and 42°C, and during the time of this case, temperatures in northern Alabama were 4.4°C to 20°C [4]. There is limited research regarding the bacteria's transmission in humans, unlike amongst fish, where the pathogen has been found to spread via direct transmission in the same water source as well as by fecal-oral route [1]. In this particular case of a child wading in a creek, it is nigh impossible to ascertain definitively whether the infection was acquired directly from local fish or related to the animals housed nearby and their waste runoff. Another possible vector that cannot be ruled out is that of the boots the patient was wearing instead of exposure to contaminated water. While such a method of transmission is not very well studied, it cannot be entirely excluded from the realm of possibility, and as such, further inquiry in this direction should be performed. It is also important to note that not all strains of *L. garvieae* are pathogenic to humans or fish [15]. While there are several methods to identify *L. garvieae*, including but not limited to proteomic, phenotypic, and molecular methods, it is important to be aware of possible misdiagnoses, as *Lactococcus* can be difficult to differentiate from *Enterococcus* [3]. Nonetheless, most research thus far has been focused on clinical isolates from fish and knowledge of the extent of *L. garvieae’s* virulence factors is limited [4].

## Conclusions

In this case report, we identify the findings found in a healthy 6-year-old male with superficial wounds contaminated with *L. garvieae*, a bacterium that is typically found to infect patients through ingestion. Making note of this patient’s lack of significant medical or family history, this patient successfully improved in health without further clinical manifestations, unlike previously reported cases. With the use of improved identification approaches and the increased awareness of the clinical relevance of *L. garvieae* as a human pathogen, infections appear to have increased in the last decade and, as such, should remain in the back of the mind of a skilled clinician when evaluating patients. The possibility remains that the incidence of *L. garvieae* infections in humans is not fully quantified, as isolated cases detected in routine laboratory work may not be published as they are identified.
